# Mite Domatia and Associated Mite Density in a North American Eastern Deciduous Forest in Michigan

**DOI:** 10.1002/ece3.71379

**Published:** 2025-04-23

**Authors:** Carolyn D. K. Graham, Lillian R. Bailey, Ashley E. Cole, Anna M. Cress, Emma Dawson‐Glass, Bailee D. Duke, Liam J. Estill, Lauren D. Jones, Gabrielle R. Leon, Samantha Molino, Nia G. Paton, Abrianna J. Soule, Christopher A. Talbot, Addison L. Yerks, Marjorie G. Weber

**Affiliations:** ^1^ Ecology and Evolutionary Biology Department University of Michigan Ann Arbor Michigan USA; ^2^ University of Michigan Biological Station University of Michigan Pellston Michigan USA

**Keywords:** acarodomatia, defense, mutualism, plant–mite interactions

## Abstract

Mite–plant defense mutualisms are among the most common defense mutualisms in the world—yet studies providing basic information on their prevalence in plant communities remain rare. Here, we systematically surveyed common woody plants in a North American deciduous forest for the presence of plant–mite mutualistic interactions. We scored 16 common woody species in a wooded natural area for the presence and number of mite domatia—small structures on the underside of plant leaves that are known to house mutualistic mites. We found that 80% of common woody species in the forest had mite domatia, the highest reported percentage of mite domatia in any survey conducted thus far. We paired our survey with a quantification of the number of mites found on each leaf and investigated the relationship between mite domatia and mite abundance within and across species. We found that plants with mite domatia had significantly more mites on their leaves than species that lacked mite domatia, and that plants with more domatia had more mites. Together, our study provides much needed systematic survey data on plant–mite mutualism prevalence in an important plant community and points to northern temperate forests as a promising system to study plant–mite mutualisms in high densities in the future.

## Graphical abstract

Here, we characterize the incidence of a widespread yet understudied mutualism in a Northern Michigan deciduous forest: that between woody plants and fungivorous or predatory mites. We found that over 80% of the common woody species present in the habitat had mite domatia, and that mite abundance was consistently correlated with domatia presence and density across species. This work answers recent calls for more systematic surveys of plant–mite mutualisms in a wide range of environments, demonstrating the pervasiveness of domatia across woody deciduous forests in temperate environments.

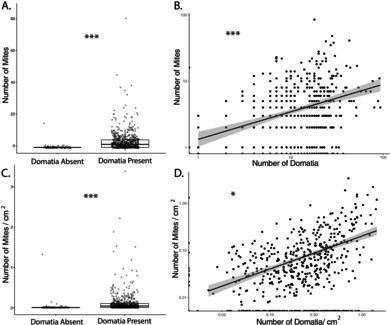

## 1 Introduction

Plants commonly engage in mutualistic interactions for defense against herbivores or pathogens and have evolved a suite of adaptations for attracting or retaining protective mutualists. Among these mutualistic defense traits, mite domatia (also known as “acarodomatia”) are one of the most evolutionarily ancient and geographically widespread (Myers et al. 2024). Mite domatia are small chambers in the vein axils on the undersides of leaves that provide housing for predacious and fungivorous mites and their eggs. In return for housing provided by the domatia (which can protect mites from desiccation and predation), mites can protect plants by consuming small herbivores and pathogens (Agrawal and Karban 1997; English‐Loeb et al. 2002; English‐Loeb et al. 2007). Unlike many other plant phenotypes, mite domatia have a clear single ecological function, allowing for straightforward links between distributional patterns and hypotheses about the evolutionary and ecological drivers of mutualism and defense. A recent study suggests a broad association between mite domatia prevalence and temperate climates, with an increase in species with mite domatia increasing with distance from the equator and decreasing with mean annual temperature and rainfall (Myers et al. 2024). This broad association, which accounted for phylogenetic relatedness, reflects a pattern of distantly related lineages of woody plants associating with mites via domatia in temperate regions. However, this large‐scale pattern emerges from trait and geographic database extrapolations rather than systematic in situ community surveys across different habitats. Although such database‐driven studies effectively reveal broad patterns across large geographic scales, they may be influenced by geographic or taxonomic sampling biases that obscure underlying biological patterns. To better understand this important defense mutualism, additional studies thoroughly scoring mite domatia presence and abundance in communities are needed.

Systematic surveys of mite domatia presence at a single location provide a standardized assessment of the prevalence of plants with this ecologically relevant adaptation in a given habitat. Several systematic surveys of mite domatia occurrence in plant communities have been conducted to date (O'Dowd and Willson 1989; Willson 1991; O'Dowd and Pemberton 1994; Pemberton 1998; Kim 2010; Kim et al. 2012; summarized in Myers et al. 2024). In each of these studies, researchers survey woody plants for the presence or absence of mite domatia on their leaves, reporting an overall percentage of these species with domatia. Together, these studies suggest high variation in domatia presence/absence across sites. Results range from the percentage of sampled woody species with mite domatia being as low as 1% in an Australian coastal forest (O'Dowd and Willson 1989), up to 69% in a South Korean forest community (Kim et al. 2012), and over 70% in an Eastern Deciduous forest of Illinois in the United States (Willson 1991). Together, the data from previous studies reveal increased abundance of mite domatia at higher latitudes, supporting the results of the broader literature survey (Myers et al. 2024).

In this paper, we characterize the proportion of species with domatia among woody plant species in a North American North Eastern Deciduous forest in Michigan, expanding the range of the communities in North America previously sampled by ~2° latitude north. Leaves of woody plants in a natural community were surveyed for the presence of mite domatia and mites, allowing us to ask the following questions: (1) What percentage of woody species in this system have mite domatia? (2) How much variation in domatia presence and number occurs within and across species? and (3) How does the number and abundance of mites on leaves vary within and across species, and is this variation predicted by the presence of mite domatia?

## 2 Methods

To characterize the domatia investment in a Northern Michigan forest, we performed a field survey of domatia and mites on deciduous woody species at the University of Michigan Biological Station (UMBS). The UMBS is located in the northern lower peninsula of Michigan (42.2789° N, 83.7345° W). All collections were made within 1 km of the UMBS main campus in wooded natural areas of primarily beech–maple forest in July of 2024. We sampled all woody species in a roughly kilometer natural area for which we could find at least three individuals (with the exception of poison ivy which was not sampled because of handling safety concerns). For each species, we haphazardly sampled five leaves per plant for up to 10 plants per species. Immediately upon collection, leaves were placed in plastic bags with a damp paper towel and stored in a cooler to keep mites and leaves from desiccating. Leaves were then transported to the laboratory and stored in plastic bags at 4°C until processing.

Within 24 h of collection, the abaxial (bottom) surface of leaves was observed under dissecting microscopes to quantify domatia and mite presence and abundance. We recorded the number of domatia on each leaf, defining a domatium as a vein axil with either a concentration of trichomes within the leaf axil that was higher than the trichome density on the rest of the leaf/veins (tuft domatia), or a flap of laminar tissue in the vein axil that creates a cavity (pocket domatia; Table 2). We systematically searched the entire abaxial surface for mites and recorded the total number of mites per leaf. After surveying fresh leaves for domatia and mite presence, leaves were labeled, pressed, and dried. Once dry, we scanned the leaves using a CanoScan 9000F Mark II scanner (Canon, USA) and quantified leaf area using ImageJ (Schneider et al. 2012).

All analyses were conducted in the R environment version 4.4.2 (R Core Team 2024). We calculated the percentage of surveyed species in the community with mite domatia as [the number of species with mite domatia present]/[16, the total number of species surveyed] multiplied by 100. We visualized the distribution of mite domatia and mite presence and abundance within and across species using ggplot (Wickham 2016). We then placed the species into a phylogenetic context by creating a phylogeny of our 16 focal species using the R package V.phylomaker with the “scenario 3” parameters (Jin and Qian 2019).

To evaluate whether variation in the abundance of mites on leaves is predicted by the presence and/or abundance of mite domatia on sampled leaves, we conducted a series of phylogenetic generalized mixed models (GLMMs) using the R package glmmADMB (Skaug 2014; Fournier et al. 2012). Because mite abundance data are counts, all models used a negative binomial distribution, which consistently performed better than a Poisson distribution (as determined by plotting residuals, comparing Q‐Q plots, and comparing AIC values of models). We report model results evaluating whether mite abundance (response variable) is impacted by domatia abundance (predictor variable) at two scales: the interspecific scale (using all data) and the intraspecific scale (individual models within each plant species). Because domatia presence (not just abundance) is variable at the across‐species scale, we also tested whether domatia presence/absence (predictor variable) was associated with mite abundance (response variable) at the interspecific scale. Finally, because the species included in our study vary considerably in the size of their leaves (Table 1) and previous studies provide mixed evidence as to the impact of leaf size on mite–domatia interactions (e.g., Grostal and O'Dowd 1994; English‐Loeb et al. 2002), we also conducted models both with and without including leaf area (as cm^2^ leaf area) as an offset at the interspecific scale. In all analyses we accounted for non‐independence of multiple samples per plant and the scientist who collected the data by including plant and observer as random effects. In the interspecific analyses, plant was nested within species, and phylogenetic relatedness was included using the corBrownian function in the package ape (Paradis and Schliep 2019) to account for non‐independence due to shared ancestry. Statistical significance was assessed using a Wald's test with alpha values less than 0.05 interpreted as significant and alphas between 0.05 and 0.1 reported as marginally significant.

**TABLE 1:** Domatia, mite, and leaf size data from the study, along with sample sizes for each species.

Species	Domatia per leaf	Mites per leaf	Leaf Area (cm^2^)	N. individuals (leaves) sampled	Intraspecific correlation results
*Acer pensylvanicum*	22.56 (8.64)	4.32 (4.01)	112.22 (56.78)	10 (50)	**0.02, Z = 2.17**, ***p* = 0.03**
*Acer rubrum*	8.02 (5.06)	2.86 (2.81)	48.23 (17.31)	10 (50)	**0.07, Z = 2.5**, ***p* = 0.012**
*Acer saccharum*	12.36 (10.59)	4.45 (7.16)	57.06 (24.47)	11 (55)	*0.04, Z = 1.66*, *p = 0.097*
*Amelanchier arborea*	0.76 (0.92)	0.54 (1.11)	17.18 (8.04)	10 (50)	N/A
*Betula papyrifera*	8.05 (2.47)	1.75 (1.85)	30.22 (12.40)	8 (40)	**0.29, Z = 2.77**, ***p* = 0.006**
*Cornus rugosa*	12.87 (3.09)	3.23 (3.65)	56.05 (15.68)	6 (30)	0.04, *Z* = 0.78, *p* = 0.44
*Fagus grandifolia*	20.84 (8.15)	3.32 (5.61)	59.45 (23.25)	10 (50)	*0.06, Z = 1.87*, *p = 0.061*
*Fraxinus americana*	27.78 (22.77)	4.4 (4.29)	140.34 (76.12)	9 (45)	0.01, *Z* = 1.64, *p* = 0.1
*Ostrya virginiana*	17.08 (3.13)	2.33 (4.16)	30.71 (9.86)	10 (51)	0.01, *Z* = 1.62, *p* = 0.11
*Populus grandidentata*	0.2 (0.95)	0.36 (2.26)	32.89 (10.39)	10 (50)	N/A
*Populus tremuloides*	0 (0)	0 (0)	15.23 (6.47)	5 (25)	N/A
*Prunus serotina*	2.1 (0.45)	0.55 (0.89)	23.93 (11.04)	4 (20)	N/A
*Quercus rubra*	5.48 (2.78)	2.43 (2.87)	69.92 (29.76)	11 (54)	0.1, *Z* = 1.49, *p* = 0.14
*Tilia americana*	36.53 (25.34)	4.13 (3.8)	91.43 (60.14)	3 (15)	*0.01, Z = 1.78*, *p = 0.075*
*Viburnum acerifolium*	14.93 (7.39)	12.2 (14.53)	24.34 (10.99)	11 (55)	**0.05, Z = 2.82**, ***p* = 0.005**
*Vitis riparia*	12.5 (7.07)	5.52 (6.13)	50.67 (18.48)	10 (50)	*0.05, Z = 1.9*, *p = 0.057*

*Note:* Numbers represent means and, in parentheses, standard deviations. In the final column, we present the results of the intraspecific test of the relationship between domatia abundance and mite abundance, with the coefficient estimate, *z* value, and *p* value. Results with *p*‐values < 0.05 (significant) are bolded and between 0.05 and 0.1 (marginally significant) are in italics. In cases where species almost entirely lacked mites and domatia and models could not be run are marked with a N/A.

## 3 Results

**FIGURE 1:**
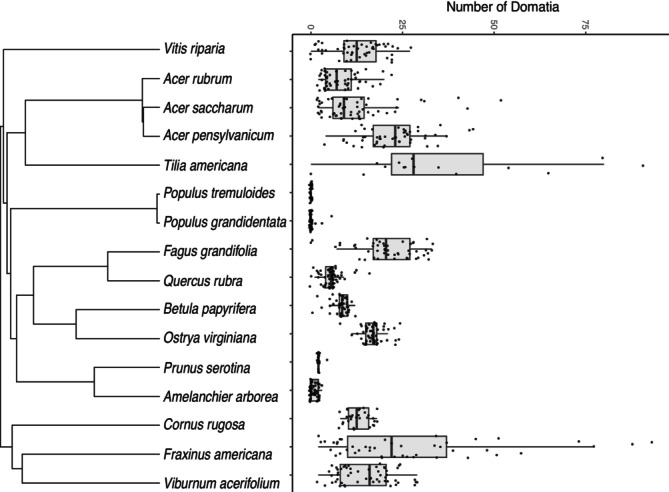
Distribution of mite domatia across woody broadleaf species in an eastern North American forest in Michigan. Each dot represents the domatia count from an individual leaf.

We scored domatia and counted mites on 690 leaves across 16 different deciduous woody species present in the eastern deciduous forest (mean plant individuals per plant species = 8.63, SD = 2.63; mean leaves per plant species = 43.13, SD = 13.13). Of the 16 species surveyed, 13 had consistent domatia presence on their leaves. The three species in our study that we classified as having absent domatia for the purpose of presence/absence analyses were *Populus tremuloides* , *P. grandidentata* , and *Amelanchier arborea* . The abundance of mite domatia varied considerably across species, with the average number of domatia per leaf for a species ranging from 0 ( *P. tremuloides* ) to 36.5 ( *Tilia americana* ) (Figure 1, Table 1). Representative images and descriptions of domatia for each species are included in Table 2.

**TABLE 2:** Descriptions of domatia morphology and representative photographs taken from pressed leaves.

Species	Common name	Photograph	Description
*Acer pensylvanicum*	Striped Maple	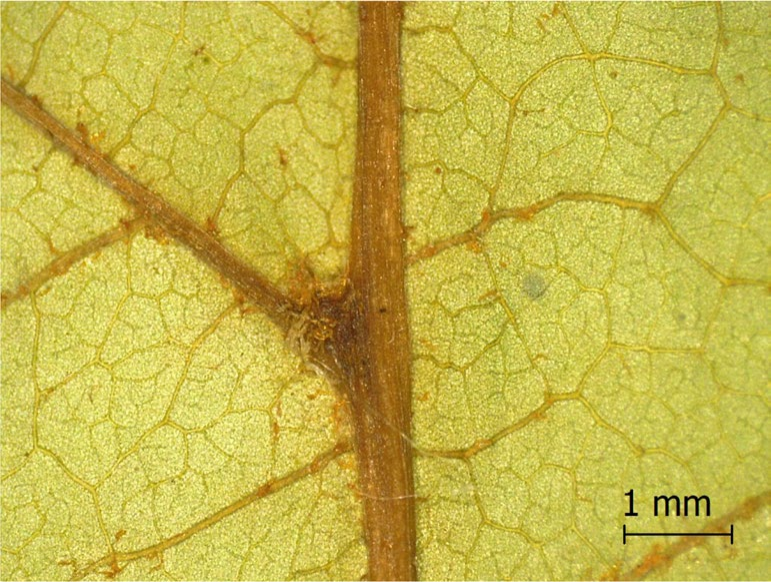	Pocket/tuft: orange or red tufts of trichomes emerging from under a cave of tissue at the vein axil.
*Acer rubrum*	Red Maple	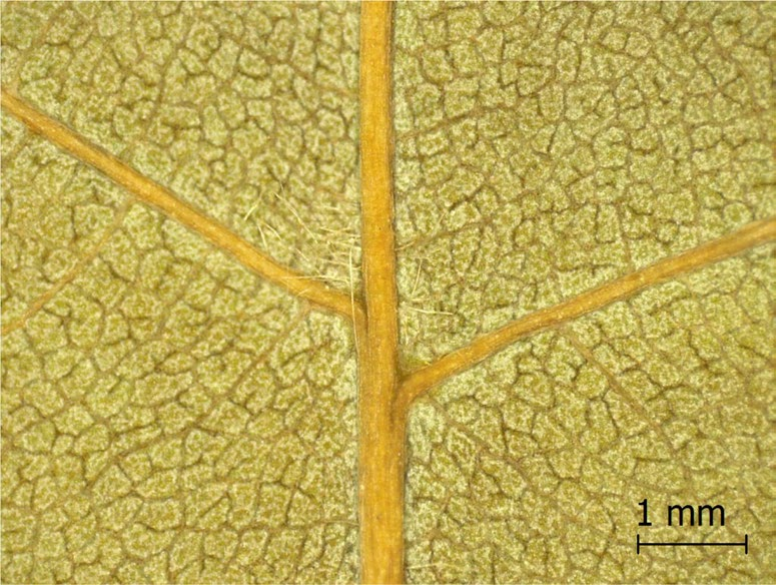	Tuft: sparse trichomes in vein axils.
*Acer saccharum*	Sugar Maple	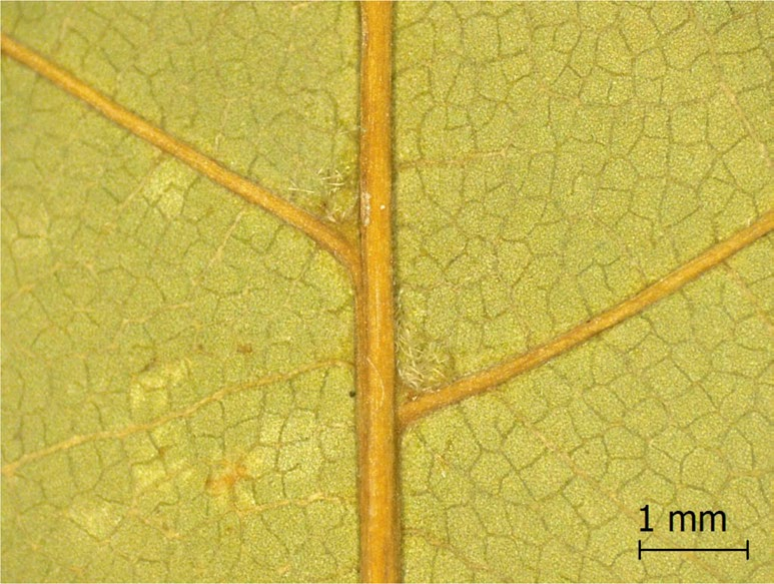	Tuft: dense tufts of trichomes in vein axils.
*Amelanchier arborea*	Serviceberry	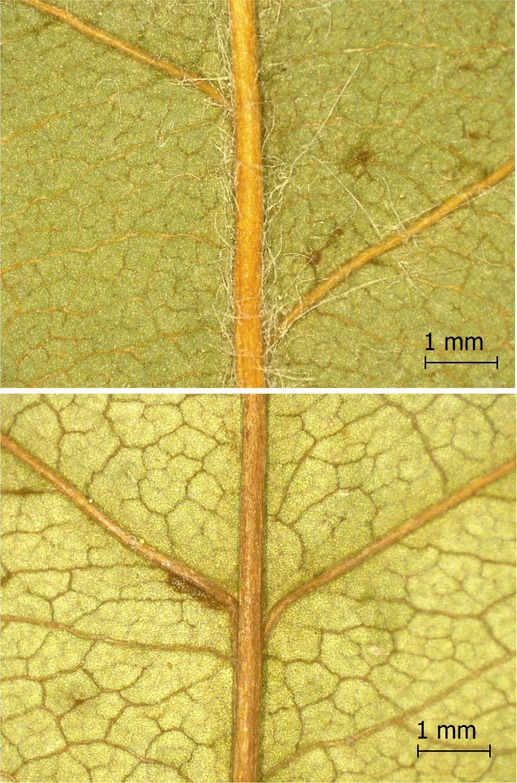	Tuft: lower midrib occasionally has dense trichomes that become sparse axially (similar to *P. serotina* ). Occasionally vein axils with sparse tufts.
*Betula papyrifera*	Paper Birch	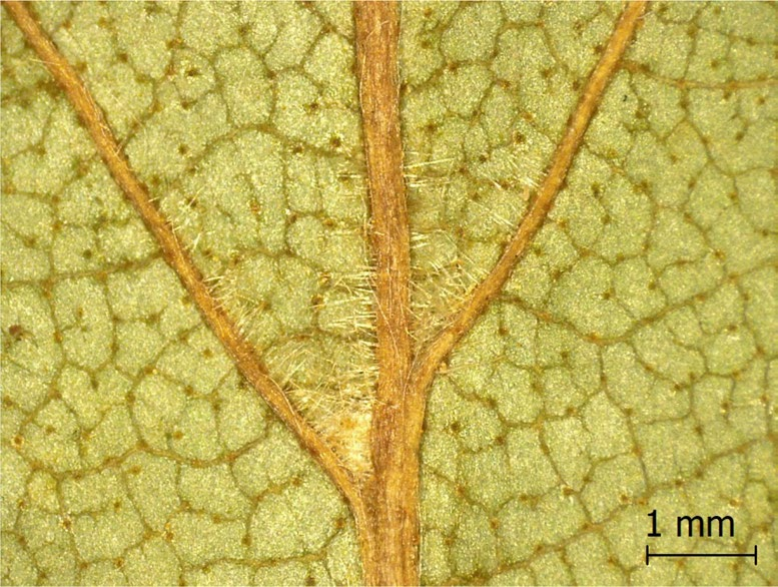	Tuft: dense tufts of trichomes in vein axils.
*Cornus rugosa*	Roundleaf Dogwood	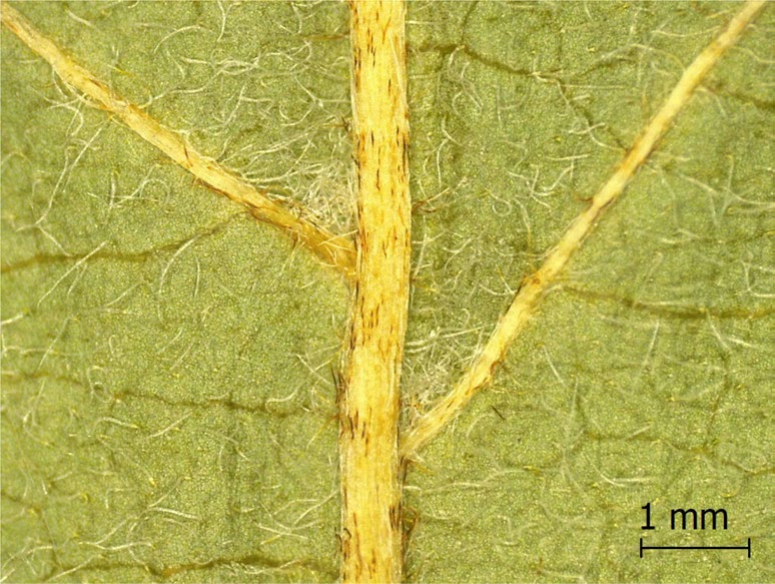	Tuft: dense tufts of trichomes in vein axils, and sparse trichomes across lower leaf surface.
*Fagus grandifolia*	American Beech	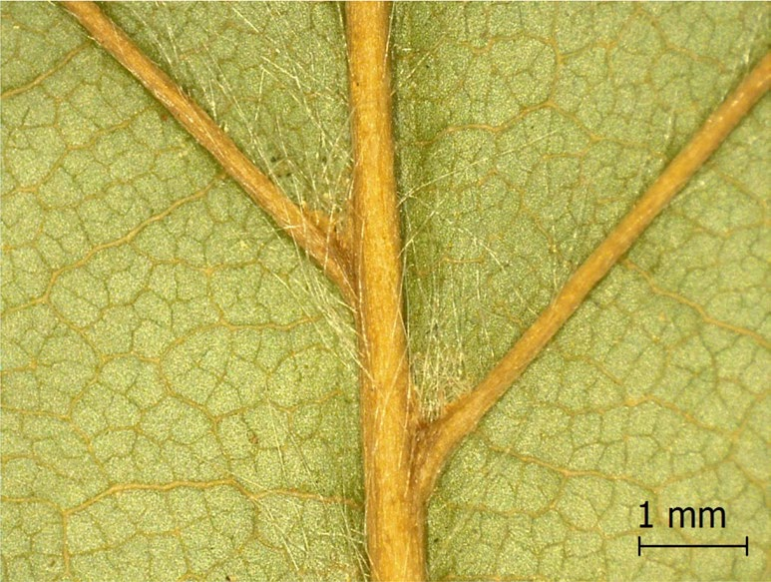	Tuft: sparse to dense patches of trichomes in vein axils and along midrib.
*Fraxinus americana*	White Ash	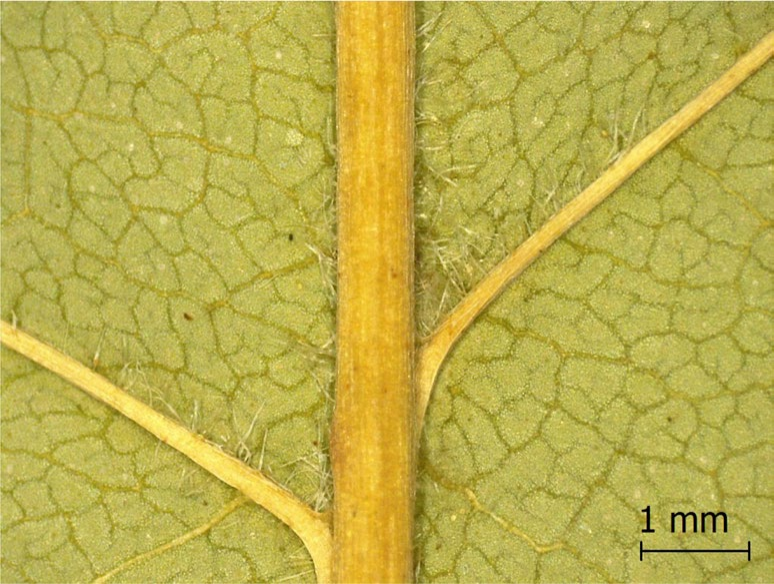	Tufts/pockets: tufts of trichomes in vein axils and along midrib, sometimes with tissue stretched over vein axil.
*Ostrya virginiana*	American Hophornbeam	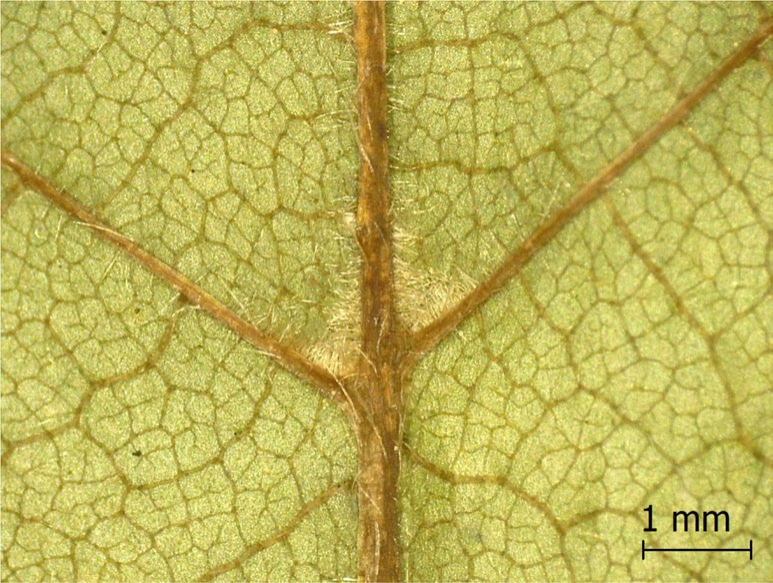	Tufts: very dense tufts of trichomes in the vein axils, sparse trichomes along midrib.
*Populus grandidentata*	Bigtooth Aspen	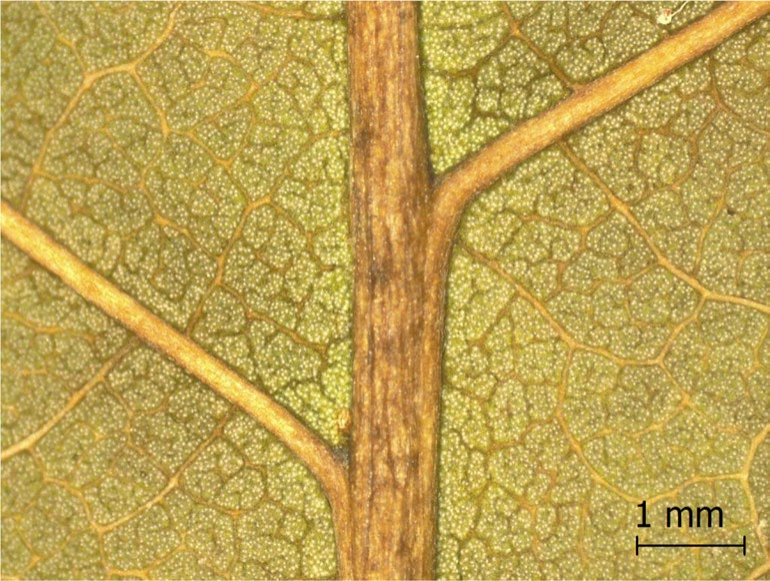	N/A
*Populus tremuloides*	Trembling Aspen	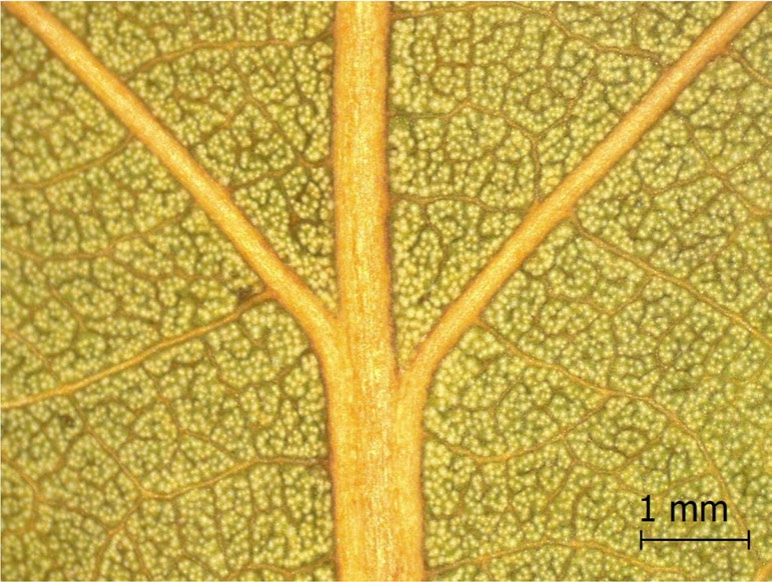	N/A
*Prunus serotina*	Black Cherry	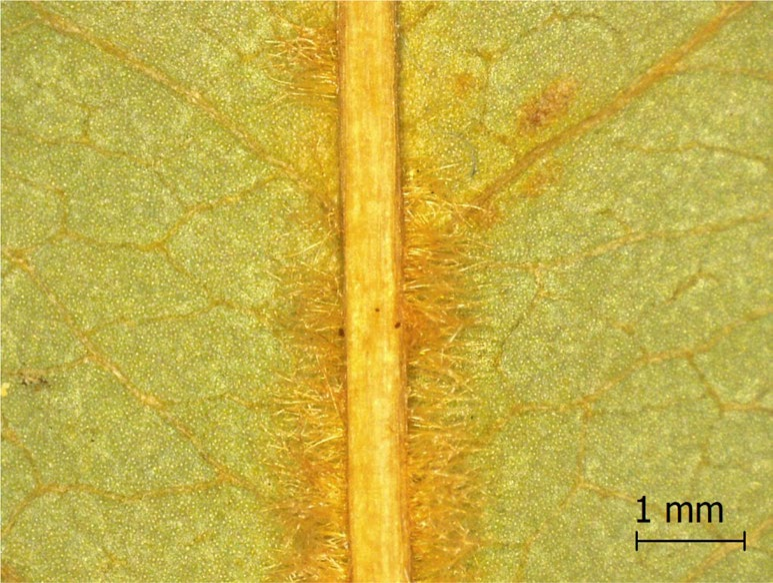	Tuft: ridge of orange trichomes along lower midrib with denser trichomes toward petiole.
*Quercus rubra*	Red Oak	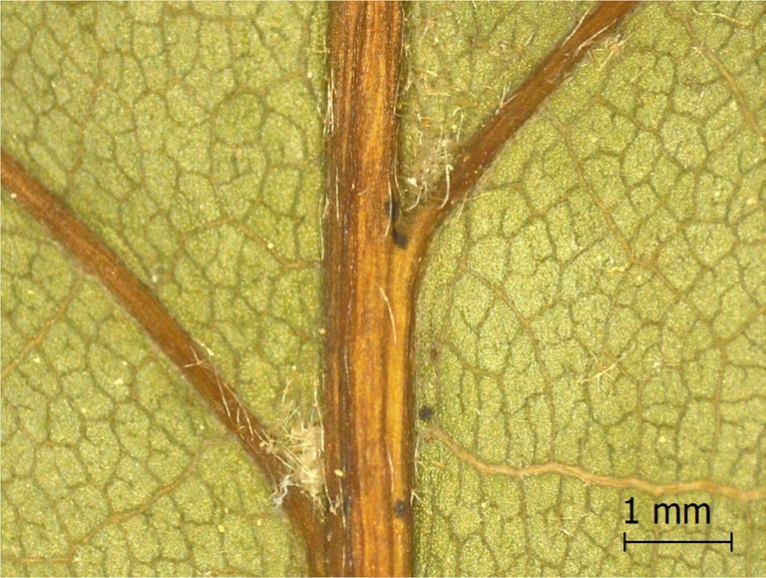	Tuft: dense patches of trichomes in vein axils.
*Tilia americana*	Basswood	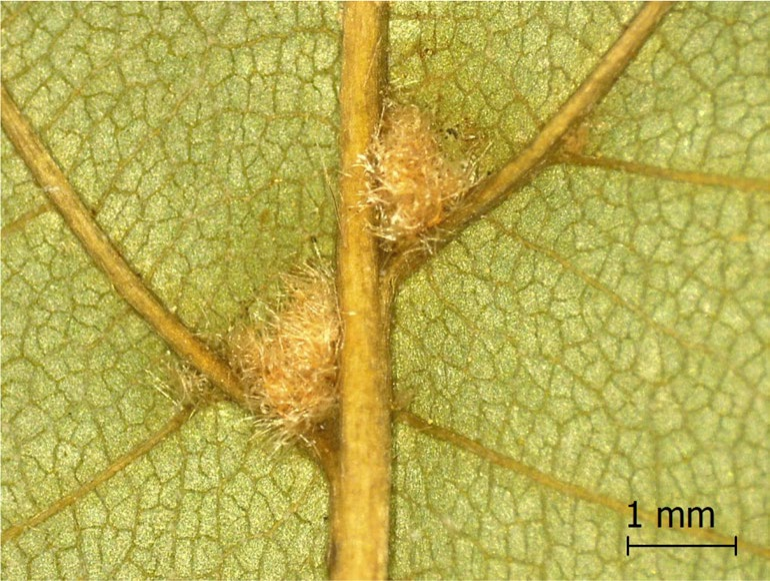	Tuft: very dense patches of trichomes in vein axils.
*Viburnum acerifolium*	Mapleleaf Viburnum/Moosewood	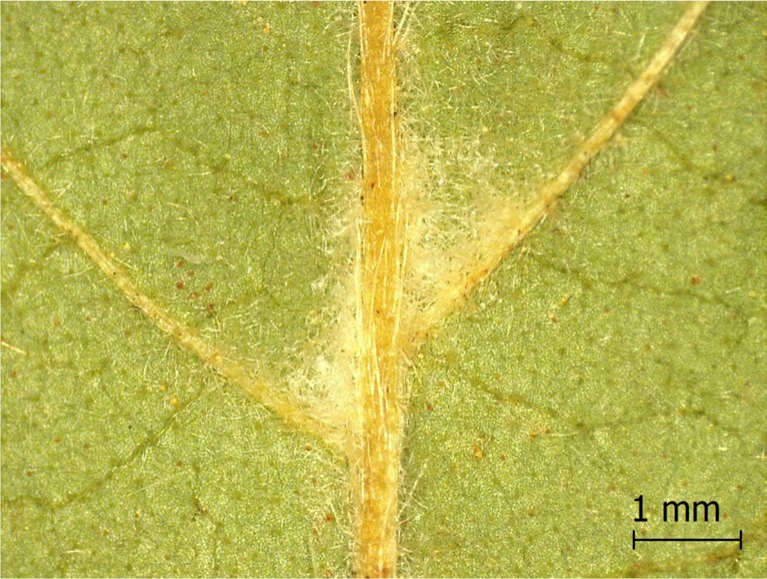	Tuft: very dense patches of trichomes in vein axils, entire leaf sparsely covered in trichomes.
*Vitis riparia*	Riverbank Grape	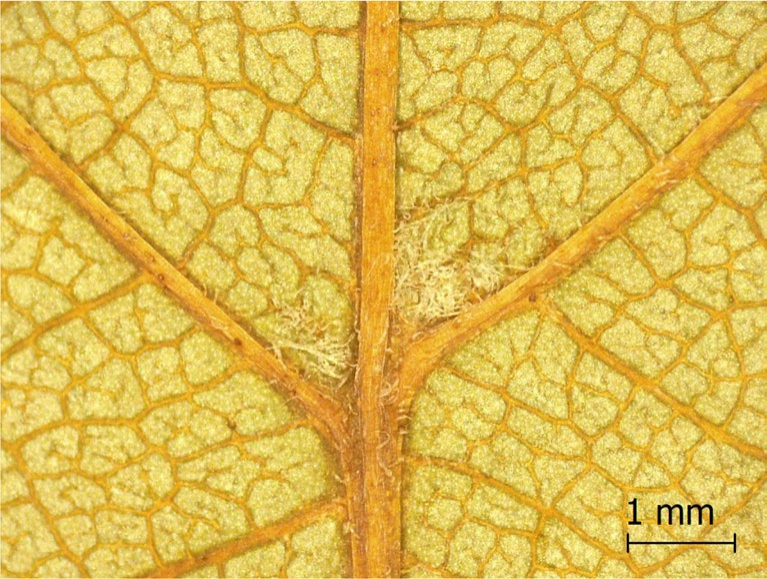	Tuft: sparse to dense patches of trichomes in vein axils, trichomes along midrib.

**FIGURE 2:**
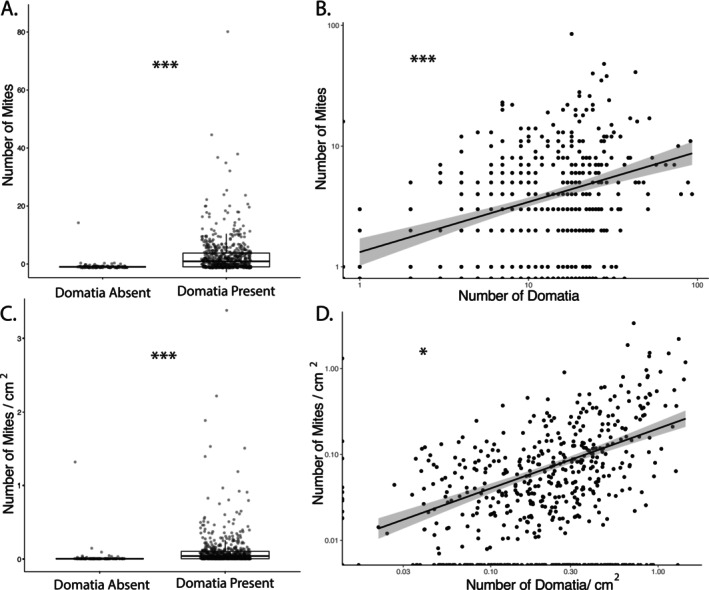
Relationship between mite domatia and the abundance and density of mites on leaves. The abundance (A) and density (C) of mites on leaves are higher on plants with mite domatia present than on plants that lack mite domatia. Leaves with more domatia have more mites, both raw abundance (B) and when controlling for leaf area (D). Note that the axes of B and D are plotted on a log scale. Significance scores from GLMM analyses are denoted with a “***” for values less than 0.01, and a “*” for marginal values, between 0.05 and 0.1.

The abundance of mites on leaves also varied considerably both within and across species, with the average number of mites per leaf for a species ranging from 0 ( *P. tremuloides* ) to 12.2 ( *Viburnum acerifolium* ) (Table 1). The abundance and density of mites on leaves were higher on plants with domatia (Figure 2A,C). Leaves with domatia had on average 17 times as many mites as leaves without domatia present (with domatia average = 4.15, SD = 6.73, without domatia = 0.23, SD = 1.56). Domatia presence was a significant predictor of mite number both with (*p* < 0.001, *z* = 6.41; Figure 2C) and without (*p* < 0.001, *z* = 7.01; Figure 2A) accounting for leaf size. The abundance of mites on leaves was also significantly positively predicted by domatia abundance where every added domatium increased the number of mites on a leaf by ~4% (*p* < 0.001, *z* = 6.40; Figure 2B). The positive relationship between mite abundance and domatia abundance was less strong (~1% increase in mite abundance with each domatium) when accounting for leaf area using an offset (marginally significant, *p* = 0.08, *z* = 1.76; Figure 2D).

**FIGURE 3:**
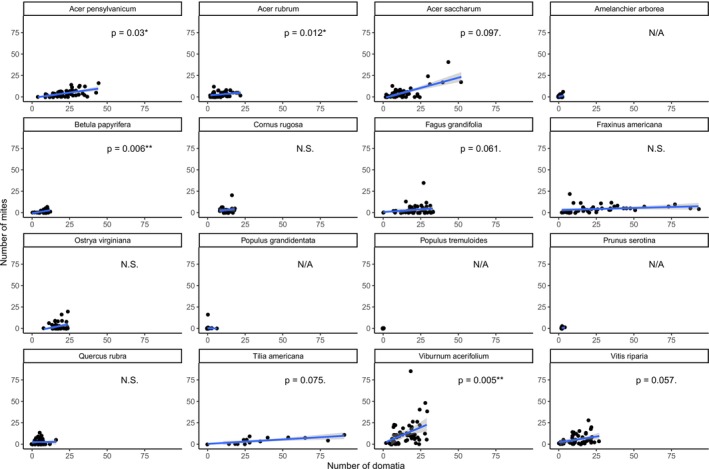
Relationship between mite abundance and domatia abundance separated by species. Significance scores from GLMM analyses are denoted with a “**” for values less than 0.01, a “*” for values between 0.01 and 0.05, and a “.” for values between 0.05 and 0.1. Note that this is different than in the significance denotation in Figure 2.

At the within‐species scale, the relationship between mite number and domatia number was variable from species to species (Table 1; Figure 3). For several species, a lack of intraspecific variation in the number of mite domatia within the species precluded the modeling of mite abundance as a function of domatia number, either because domatia were entirely or almost entirely absent across samples (*Amelanchier arborea, Populus grandidentata, Populus tremuloides*), or because all individuals of the species have the same number of domatia (*Prunus serotina*, which consistently have two domatia). For the species for which mite and domatia numbers were sufficiently variable to model, the number of domatia significantly positively predicted mite abundance in four cases (*Acer pensylvanicum, Acer rubrum, Betula papyrifera* and *Viburnum acerifolium* ), whereas four species had only marginally significant positive relationships (*Acer saccharum, Fagus grandifolia, Tilia americana, and Vitis riparia)*, and four had no significant relationship (*Cornus rugosa, Fraxinus americana, Ostrya virginiana*, and *Quercus rubra)*.

## 4 Discussion

Documenting the distribution of ecologically important traits within communities is a critical foundation for studies of the causes and consequences of ecological variation. Here, we systematically surveyed common woody plants in a North American deciduous forest to quantify the distribution of mite domatia, a relatively understudied plant phenotype that mediates an ecologically important mutualism. Recent calls for systematic surveys of mite domatia (Myers et al. 2024) in communities highlight the need for standardized quantifications of this ecologically relevant adaptation. We found that over 80% of common woody species had considerable presence of mite domatia on their leaves, the highest reported density of mite domatia in a community survey to date (Myers et al. 2024). We also found that plants with mite domatia had considerably higher abundance and density of mites on their leaves, that leaves with more domatia had more mites, and that these patterns held both within and across species. This work points to the northern forests of Michigan in the United States as a particularly promising system to study mite–plant mutualisms, and more generally reinforces recent studies highlighting temperate regions as particularly dense in mite–plant mutualisms (Myers et al. 2024).

Our study adds to the literature describing mite domatia prevalence at the community scale (O'Dowd and Willson 1989; Willson 1991; O'Dowd and Pemberton 1994; Pemberton 1998; Kim 2010; Kim et al. 2012; summarized in Myers et al. 2024). Together, these surveys create a picture of the density and distribution of mite domatia across different habitat types. However, the lack of standardized survey approaches makes comparing specific data across studies challenging. Future work repeating standardized methods across spatial and environmental gradients will be particularly fruitful in hypothesis testing, especially where environmental factors are hypothesized to impact the ecology and evolution of defense or mutualisms. The methods used in this paper could serve as a model for future surveys. In particular, standardized surveys testing for increased domatia prevalence in temperate, cold, and wet habitats can corroborate patterns from recent analyses based primarily on projections from trait and geographical databases (Myers et al. 2024).

The observed relationship between domatia and mite density on leaves in this study recapitulates results from previous work linking the phenotype with enhanced mite populations (reviewed in O'Dowd and Willson 1991; Romero and Benson 2005). Within species, experimental studies have demonstrated that removing or blocking domatia reduces beneficial mites on leaves, while artificially adding domatia‐like structures increases mite numbers (Grostal and O'Dowd 1994; Romero and Benson 2004; Walter and O'Dowd 1992; Grostal and O'Dowd 1994; English‐Loeb et al. 2002; A. Agrawal 1997; A. A. Agrawal and Karban 1997; Graham et al. 2022). Furthermore, similar surveys have consistently found that species with domatia harbor larger populations of beneficial mites on leaves compared with species without domatia (e.g., O'Dowd and Pemberton 1998; Rozario 1995; Walter and O'Dowd 1992; O'Dowd and Willson 1997). Our study adds to the growing body of experimental manipulations and comparative surveys providing evidence that domatia are a key adaptation for promoting mite populations across diverse plant lineages. Notably, although we found a strong positive correlation between mite abundance and domatia abundance across woody species in this forest, the presence of significant relationships between these parameters within individual species was variable. The reasons for the lack of correlations between mites and domatia within some woody species are unclear; thus, investigations examining determinants of host quality for mites across domatia‐bearing species are important future work.

Our study lays the groundwork for future research on the structure of mite communities within and across leaves of woody plant species in deciduous forests of Michigan. Although our study demonstrated a relationship between domatia and mite abundance, one limitation was the lack of information on the identity of the mite species. Accurate identification of mite taxa is notoriously challenging, and in this case identifying this number of mite species was beyond our resources and thus the scope of our study. Although several studies have attempted taxonomic identification of mites in domatia surveys and found largely predatory and fungivorous taxa (e.g., O'Dowd and Pemberton 1998; Rozario 1995; Walter and O'Dowd 1992; O'Dowd and Willson 1997), a promising area of future research would be to build on this work by integrating morphological and molecular identification approaches. Such work would allow for larger‐scale quantification of mite community patterns, investigating patterns of hidden diversity and drivers of genetic relatedness of mites across forests. For example, future work asking whether plant leaf phenotypic similarity, physical proximity, or phylogenetic relatedness dictates similarity in mite communities would be particularly impactful.

## 5 Conclusion

Plants have evolved remarkable traits to facilitate mutualistic relationships, developing adaptations to attract and sustain protective partners. Here, we systematically surveyed a North American deciduous forest for one of the most common and ancient defense mutualism phenotypes: mite domatia. Our study answers calls for additional systematic surveys of the presence of mite domatia and mites on leaves. The findings build on work linking domatia and mite abundance, and point to northern temperate forests as a promising system for studying mite–plant mutualisms in high densities in the future.

## Author Contributions

**Carolyn D. K. Graham:** conceptualization (lead), data curation (lead), formal analysis (equal), investigation (lead), methodology (lead), project administration (lead), supervision (equal), visualization (equal), writing – original draft (equal), writing – review and editing (equal). **Lillian R. Bailey:** conceptualization (supporting), investigation (supporting), methodology (supporting), writing – review and editing (supporting). **Ashley E. Cole:** conceptualization (supporting), investigation (supporting), methodology (supporting), writing – review and editing (supporting). **Anna M. Cress:** conceptualization (supporting), investigation (supporting), methodology (supporting), writing – review and editing (supporting). **Emma Dawson‐Glass:** conceptualization (supporting), investigation (supporting), methodology (supporting), writing – review and editing (supporting). **Bailee D. Duke:** conceptualization (supporting), investigation (supporting), methodology (supporting), writing – review and editing (supporting). **Liam J. Estill:** conceptualization (supporting), investigation (supporting), methodology (supporting), writing – review and editing (supporting). **Lauren D. Jones:** conceptualization (supporting), investigation (supporting), methodology (supporting), writing – review and editing (supporting). **Gabrielle R. Leon:** conceptualization (supporting), investigation (supporting), methodology (supporting), writing – review and editing (supporting). **Samantha Molino:** conceptualization (supporting), investigation (supporting), methodology (supporting), writing – review and editing (supporting). **Nia G. Paton:** conceptualization (supporting), investigation (supporting), methodology (supporting), writing – review and editing (supporting). **Abrianna J. Soule:** conceptualization (supporting), investigation (supporting), methodology (supporting), writing – review and editing (supporting). **Christopher A. Talbot:** conceptualization (supporting), investigation (supporting), methodology (supporting), writing – review and editing (supporting). **Addison L. Yerks:** conceptualization (supporting), investigation (supporting), methodology (supporting), writing – review and editing (supporting). **Marjorie G. Weber:** conceptualization (equal), data curation (equal), formal analysis (equal), funding acquisition (lead), investigation (equal), methodology (equal), project administration (equal), supervision (equal), visualization (equal), writing – original draft (equal), writing – review and editing (equal).

## Conflicts of Interest

The authors declare no conflicts of interest.

## Supporting information


Data S1.

## Data Availability

All the required data are uploaded as Supporting Information.
